# Low-frequency electroacupuncture suppresses focal epilepsy and improves epilepsy-induced sleep disruptions

**DOI:** 10.1186/s12929-015-0145-z

**Published:** 2015-07-07

**Authors:** Pei-Lu Yi, Chin-Yu Lu, Shuo-Bin Jou, Fang-Chia Chang

**Affiliations:** Department of Veterinary Medicine, School of Veterinary Medicine, National Taiwan University, No. 1, Sec. 4., Roosevelt Road, Taipei, 106 Taiwan; Department of Sports, Health & Leisure, College of Tourism, Leisure and Sports, Aletheia University, Tainan Campus, Tainan, Taiwan; Department of Neurology, Mackay Memorial Hospital and Mackay Medical College, Taipei, Taiwan; Graduate Institute of Brain & Mind Sciences, College of Medicine, National Taiwan University, Taipei, Taiwan; Graduate Institute of Acupuncture Science, College of Chinese Medicine, China Medical University, Taichung, Taiwan

**Keywords:** Amygdala, Electroacupuncture, Epilepsy, Feng-Chi (GB20), Opioid receptors, Sleep

## Abstract

**Background:**

The positive effects of acupuncture at Feng-Chi acupoints on treating epilepsy and insomnia have been well-documented in ancient Chinese literature. However, there is a lack of scientific evidence to elucidate the underlying mechanisms behind these effects. Our previous study demonstrated that high-frequency (100 Hz) electroacupuncture (EA) at Feng-Chi acupoints deteriorates both pilocarpine-induced focal epilepsy and sleep disruptions. This study investigated the effects of low-frequency (10 Hz) EA on epileptic activities and epilepsy-induced sleep disruptions.

**Results:**

In rats, the Feng-Chi acupoint is located 3 mm away from the center of a line between the two ears. Rats received 30 min of 10 Hz EA stimuli per day before each day’s dark period for three consecutive days. Our results indicated that administration of pilocarpine into the left CeA at the beginning of the dark period induced focal epilepsy and decreased both rapid eye movement (REM) sleep and non-REM (NREM) sleep during the consequent light period. Low-frequency (10 Hz) EA at Feng-Chi acupoints suppressed pilocarpine-induced epileptiform EEGs, and this effect was in turn blocked by naloxone (a broad-spectrum opioid receptor antagonist), but not by naloxonazine (a μ-receptor antagonist), naltrindole (a δ-receptor antagonist) and *nor*-binaltorphimine (a κ-receptor antagonist). Ten Hz EA enhanced NREM sleep during the dark period, and this enhancement was blocked by all of the opioid receptor antagonists. On the other hand, 10 Hz EA reversed pilocarpine-induced NREM suppression during the light period, and the EA’s effect on the sleep disruption was only blocked by naloxonazine.

**Conclusions:**

These results indicate that low-frequency EA stimulation of Feng-Chi acupoints is beneficial in improving epilepsy and epilepsy-induced sleep disruptions, and that opioid receptors in the CeA mediate EA’s therapeutic effects.

## Background

Epilepsy is one of the most common and devastating neurological disorders. Seventy percent of epilepsy patients can be controlled by current anti-epileptic drugs (AEDs); however, seizures recur in 30 % of patients who do not respond to any of the first-line AEDs despite administration of the optimized dosage [1]. Epilepsy patients experience more daytime somnolence than that of control patients [2], and children with epilepsy may suffer from poor quality of sleep, anxiety about sleep and sleep-disordered breathing [3]. Our previous studies have demonstrated that epilepsy occurring at different zeitgeber times results in different sleep disruptions by altering either the homeostatic factors or circadian rhythm of the sleep regulations in rats [4, 5]. Sleep disturbance notably deteriorates and worsens the progression of epilepsy [6]. Therefore, if a therapy both suppresses epilepsy and improves sleep disruptions, it becomes the optimal therapy for seizure control. Several alternative therapies, such as vagus nerve stimulation (VNS) [7, 8] and deep brain stimulation (DBS) [9], have been considered for treating refractory epilepsy. Our previous study elucidated that electrical stimulation of the left anterior thalamic nucleus with a high-frequency and low-intensity current reduces the rate of pilocarpine-induced epilepsy in rats [9]. Acupuncture is another option for seizure suppression. Indications of acupuncture of the Feng-Chi (GB20) acupoints have been documented in the ancient Chinese literature Lingshu Jing (Classic of the Miraculous Pivot) for its use in suppressing epilepsy and treating insomnia. Acupuncture may suppress seizures [10, 11] and improve insomnia [12, 13] through the activation of vagus nerve, which subsequently activates the nucleus of the tractus solitaries (NTS) and projects rostrally to the hypothalamus, amygdala, dorsal raphe nucleus, nucleus ambiguus, parabrachial nucleus, and thalamus [14–20].

Our previous studies demonstrated that the administration of pilocarpine into the left central nucleus of amygdala (CeA) induces focal epilepsy [21]. High-frequency (100 Hz) EA stimuli of the bilateral Feng-Chi acupoints, in which a 30-min EA stimulation was performed prior to the dark period of the light-dark cycle three consecutive days before the administration of pilocarpine, did not suppress the pilocarpine-induced epileptiform electroencephalograms (EEGs); rather, it further increased the duration of epileptiform EEGs in rats [21]. Pilocarpine-induced epilepsy further decreases both rapid eye movement (REM) sleep and non-REM (NREM) sleep [22]. High-frequency EA stimuli of Feng-Chi acupoints deteriorate pilocarpine-induced sleep reduction [22]. These results suggest that high-frequency EA stimuli of Feng-Chi acupoints exhibit no benefit in protecting against pilocarpine-induced epilepsy and sleep disruptions. It has been demonstrated that high frequency stimulation of the vagus nerve causes desynchronized EEG activity in the cortex and blocks sleep spindles during slow wave sleep (SWS) [23, 24]. Stimulation of the NTS at low frequencies (1-16 Hz) produces EEG synchronization, whereas high frequency (>30 Hz) stimulation results in EEG desynchronization [25]. In accordance with the aforementioned observations and our results that showed that different frequencies of EA stimulation on the same acupoint activate different neural mechanisms [12, 13], we hypothesized that low frequency (10 Hz) EA stimulation possesses different mechanisms than that of high frequency (100 Hz) EA and exhibits effects in epilepsy suppression and the improvement of epilepsy-induced sleep disturbances. The amygdala receives the afferent projection from the NTS [17, 26]. Altering the NTS activity changes the dynorphin gene expression in the amygdala [27]. It has been demonstrated that intracerebroventricular (ICV) administration of dynorphin suppresses electroconvulsive shock- and kindling-induced seizure [28, 29]. The temporal lobe epilepsy increases opioid receptors in the temporal cortex in humans [30], which may mediate the anticonvulsant effects to limit the spread of electrical activity from other temporal lobe structures [29, 31]. Based upon these observations, we further proposed that stimulation of Feng-Chi acupoints activates the vagus nerve and NTS, which subsequently modulate the opioid receptors in the amygdala to achieve its effects in suppressing focal epilepsy and blocking epilepsy-induced sleep disruptions.

## Methods

### Pharmacological agents

Stock solutions of a broad-spectrum opioid antagonist (naloxone hydrochloride (Tocris, Bristol, UK)), a μ-receptor antagonist (naloxonazine dihydrochloride (Tocris)), a δ-receptor antagonist (naltrindole hydrochloride (Tocris)) and a κ-receptor antagonist (*nor*-binaltorphimine dihydrochloride (Tocris)) were dissolved in pyrogen-free saline (PFS). Pilocarpine (1 mg/μl, Sigma-Aldrich, St. Louis, MO, USA) was also dissolved in PFS. The stock solutions were stored at 4 °C until use. Our previous results and others have indicated that the appropriate microinjection dosage for naloxonazine, naltrindole and *nor*-binaltorphimine to selectively block μ-, δ- and κ-opioid receptors, respectively, without interaction with other opioid receptor subtypes, is within 20 μg [12, 13, 32, 33]. In the current study, naloxone, naloxonazine, naltrindole and *nor*-binaltorphimine were microinjected at a dose of 10 μg/μl, which according to our previous studies efficiently exhibits pharmacological blockade [12, 13]. The total volume for each injection was 1 μl and the duration of injection was 3 to 5 min. Our previous study has demonstrated that microinjection of 1 μl solution into the CeA does not cause CeA lesion [34].

### Animals

Male Sprague-Dawley rats (250 - 300 g; National Laboratory Animal Breeding and Research Center, Taiwan) were used in this study. Rats were anesthetized by intraperitoneal injection with 50 mg/kg Zoletil® (Virbac, Carros, France), which contains tiletamine (an NMDA receptor antagonist) and zolazepam (a tranquilizer). Rats were surgically implanted with three EEG screw electrodes as previously described [35] as well as a microinjection guide cannulae directed into the left CeA (AP, 2.8 mm from bregma; ML, 4.2 mm; DV, 7.8 mm relative to bregma). The coordinates were adopted from the Paxinos and Watson rat atlas [36]. Two screw EEG electrodes were placed over the left frontal and parietal lobes of cortices, and a third EEG electrode was placed over the right cerebellum and served to ground the animal to reduce signal artifacts. Insulated leads from EEG electrodes were routed to a Teflon pedestal (Plastics One, Roanoke, VA, USA). The Teflon pedestal was then cemented to the skull with dental acrylic (Tempron, GC Co., Tokyo, Japan). The incision was treated topically with polysporin (polymixin B sulfate – bacitracin zinc) and the animals were allowed to recover for seven days prior to the initiation of experiments. Rats were housed separately in individual recording cages in the isolated room, in which the temperature was maintained at 23 ± 1 °C and the light:dark (L:D) rhythm was controlled in a 12:12 h cycle (40 Watt x 4 tubes illumination). Food (5001 rodent diet, LabDiet) and water were available *ad libitum*. All procedures performed in this study were approved by the National Taiwan University Animal Care and Use Committee.

### Experimental protocol

On the 2^nd^ postsurgical day, these rats were connected to the recording apparatus (see below) via a flexible tether. As such, rats were allowed relatively unrestricted movement within their own cages. One week after rats had adapted to the 12:12-h L:D cycle after surgery, 24-h undisturbed baseline recordings of EEGs and sleep-wake activity were obtained beginning at dark onset on the 1^st^ day of recording in rats from all groups. Eight groups of rats were used in the study as follows. Rats in group 1 (n = 6) received a 30-min 10 Hz EA stimulation of the bilateral Feng-Chi acupoints per day, beginning 30 min before the dark period and performed over three consecutive days (the *EA* group). EEGs and sleep-wake activities were recorded right after the end of the last period of EA stimuli and lasted for 24 h. Rats in group 2 (n = 6) were administered with pilocarpine in the left CeA, and EEGs and sleep-wake activities were recorded beginning from the dark onset of the L:D cycle (the *pilocarpine* group). In group 3 (n = 6), rats received the same EA stimulation protocol as those rats in the group 2 and were respectively administered with PFS and pilocarpine into the CeA before and after the last period of EA stimulation (the *PFS + EA + pilocarpine* group). Rats in group 4 (n = 6) were used to determine the effects of the opioid receptor antagonist, naloxone, on the 10 Hz EA-induced alterations in the epileptiform EEGs and sleep alterations (the *naloxone + EA + pilocarpine* group). Rats in group 5 (n = 6), 6 (n = 6), and 7 (n = 6) were respectively used to depict the effects of the μ-receptor antagonist (naloxonazine, the *naloxonazine + EA + pilocarpine* group), δ-receptor antagonist (naltrindole, the *natrindole + EA + pilocarpine* group) and κ-receptor antagonist (*nor*-binaltorphimine, the *nor-binaltorphimine + EA + pilocarpine* group) on the 10 Hz EA-induced alterations of the epileptiform EEGs and sleep activities. Rats in groups 4-7 received a similar protocol to that of the group 3, except that naloxone (10 μg/μl), naloxonazine (10 μg/μl), naltrindole (10 μg/μl) and *nor*-binaltorphimine (10 μg/μl) were administered into the CeA before the last period of EA stimulation in group 4, 5, 6 and 7, respectively. Rats in group 8 (n = 6) had a similar protocol to that of the group 3, except that rats received the sham EA stimulation (the *sham EA* group, the sham EA stimulation described later). When 10 Hz EA was given (see later), all rats were lightly anesthetized with 25 mg/kg of zoletil (half of the dose which we used for surgery), and would wake up after 30 to 35 min. A 30-min period of EA stimulation was administered before the onset of the dark period every day and was applied in three consecutive days. The anesthetization was given 30 min prior to the dark period onset and lasted for 30 min. The 10 Hz EA stimulus was delivered via the bilateral insertion of stainless needles (32 gauge x 1”, Shanghai Yanglong Medical Articles Co.) on Feng-Chi (GB20) acupoints at a depth of 2 mm. The stimulus consisted of a train of biphasic pulses (150 μs duration each) of 10 Hz with intensity of 1 mA, and was delivered by Functions Electrical Stimulator (Trio 300, I.T.O., Japan). The location of Feng-Chi acupoints in the rats is anatomically similar to that in humans. The Feng-Chi acupoints (GB 20) is located in the depression between the upper portion of m. sternocleidomastoideus and m. trapezius in humans. In rats, the Feng-Chi acupoint is located 3 mm away from the center of a line between the two ears [37]. Sham EA was performed with the stimulation of a non-acupoint located at the ventral conjunction between the forelimb and the trunk as previously described [38]. Rats, anesthetized by zoletil, received the same electrical stimuli, including the same intensity and frequency, but the stimulation site was not the location of any acupoint.

### Apparatus and recording

Signals from the EEG electrodes were fed into an amplifier (Colbourn Instruments, Lehigh Valley, PA; model V75-01). The EEG was amplified (factor of 5,000) and analog bandpass filtered between 0.1 and 40 Hz (frequency response: ±3 dB; filter frequency roll off: 12 dB/octave). Gross body movements were detected by custom-made infrared-based motion detectors (Biobserve GmbH, Germany), and movement activity was converted to a voltage output, which was digitized and integrated into 1-s bins. These conditioned signals (EEGs and gross body movements) were subjected to analog-to-digital conversion with 16-bit precision at a sampling rate of 128 Hz (NI PCI-6033E; National Instruments, Austin, TX). The digitized EEG waveform and integrated values for body movement were stored as binary computer files pending subsequent analyses.

Postacquisition determination of the vigilance state was done by visual scoring of 12-s epochs using custom software (ICELUS, Mark R. Opp) written in LabView for Windows (National Instruments). The animal’s behavior was classified as either NREM sleep, REM sleep or waking based on previously defined criteria [35]. Briefly, NREM sleep is characterized by large-amplitude EEG slow waves, high power density values in the delta frequency band (0.5 – 4.0 Hz), and lack of gross body movements. During REM sleep, the amplitude of the EEG is reduced, the predominant EEG power density occurs within the theta frequency (6.0 – 9.0 Hz) and there are phasic body twitches. During waking, the rats are generally active. There are protracted body movements. The amplitude of the EEG is similar to that observed during REM sleep, but power density values in the delta frequency band are generally greater than those in theta frequency band.

Postacquisition determinations of the onset and the duration of the EEG seizure occurrence were done by the visual scoring using AxoScope 10 Software (Molecular Devices, Sunnyvale, CA, USA). We defined EEG documented seizures as the visualization of epileptiform spikes with amplitudes greater than 2 mV appearing in discharges lasting for at least 30 s [9].

### Statistical analyses for experiment protocol

All values acquired from the EEG recordings were presented as a mean ± SEM for the indicated sample sizes. Unpaired student t-tests for the duration of epileptiform EEGs were performed to analyze and compare the difference between groups. Values acquired from sleep-wake recordings were also indicated as a mean ± SEM. One-way ANOVA for the duration of each vigilance state (NREM sleep, REM sleep, WAKE) was performed, comparing the effects of different manipulations between groups across a certain of time block. If statistically significant differences were detected, a Fisher’s post-hoc comparison was made to determine which values during experimental conditions deviated from those obtained from the control condition. An α level of p < 0.05 was considered to indicate a statistically significant difference.

## Results

### The effect of 10 Hz EA on pilocarpine-induced focal epilepsy

**Fig. 1:**
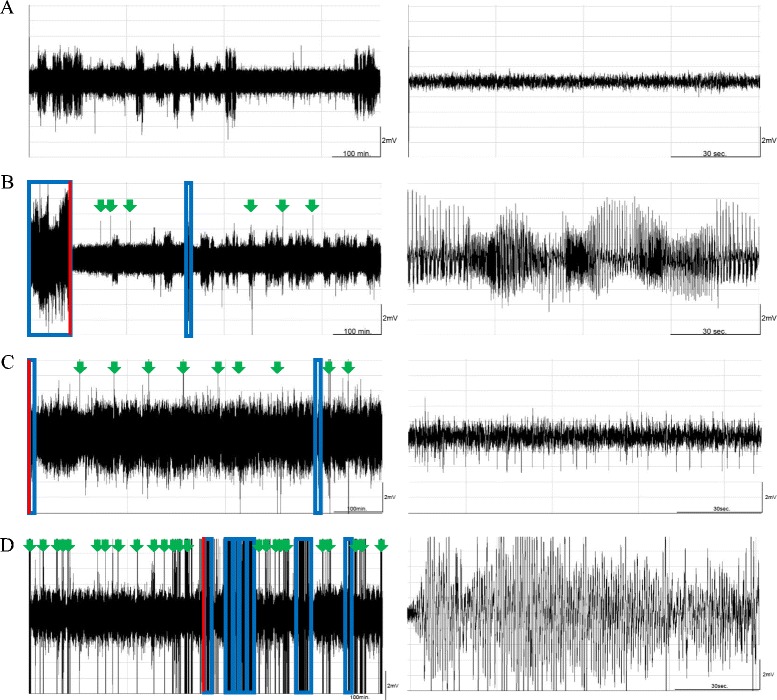
The effect of 10 Hz EA stimulation of bilateral Feng-Chi acupoints and naloxone on epileptic activities. Panels **a**, **b**, **c** and **d** respectively depict the EEG signals recorded from the naïve rats, the *pilocarpine* group, the *PFS + EA + pilocarpine* group and the *naloxone + EA + pilocarpine* group, beginning from the dark onset of the dark period. Pilocarpine was administered at time 0 in the left panels of **b**, **c** and **d**. The blue boxes represent the epileptiform EEGs. Red lines indicate the extracted time points for the expanded time-scale figures in the right panels. Green arrowheads are the artifacts. The larger amplitudes, with EEG signals less than 2 mV that appeared in panels **a**, were delta waves, which represent the state of slow wave sleep

**Fig. 2:**
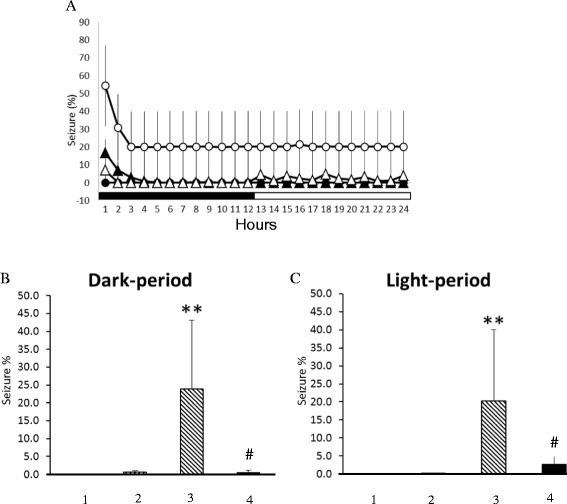
A summary of 10 Hz EA stimulation of bilateral Feng-Chi acupoints on epileptic activities. In panel **a**, the black circles represent the values obtained from the naïve group, the black triangles depict the results of the *EA* group, the white circles demonstrate the values obtained from the *pilocarpine* group, and the white triangles indicate the data acquired from the *PFS + EA + pilocarpine* group. The black and white horizontal bars represent the 12-h dark and 12-h light period of the 12:12 h light dark cycle. Panel **b** depicts the results obtained from the dark period and panel **c** demonstrates the data acquired from the light period. Bars 1-4 spanning from the left to the right in panels **b** and **c** represent the results obtained from the naïve rats, the *EA* group, the *pilocarpine* group, and the *PFS + EA + pilocarpine* group, respectively. **: p < 0.01 vs. the naïve rats; #: p < 0.01 vs. the *pilocarpine* group

We determined the effect of 10 Hz EA of the bilateral Feng-Chi acupoints on the epileptiform EEG activities induced by pilocarpine. No epileptic activity was recorded in the naïve rats without any manipulation (Fig. 1a & Fig. 2a), whereas administration of pilocarpine into the CeA induced epileptiform EEG (Fig. 1b & Fig. 2). We found that 10 Hz EA of bilateral Feng-Chi acupoints slightly induced epileptiform EEGs in the first hour after EA stimuli (Fig. 2a), which is consistent with the previous observation of epileptiform EEGs produced by high-frequency (100 Hz) EA stimuli [21]. However, 10 Hz EA stimuli significantly blocked pilocarpine-induced epileptic EEG activities (Fig. 1c, Fig. 2). The average time epileptic activities were presented in the *PFS + EA + pilocarpine* group was significantly reduced from 23.9 ± 19.1 % obtained after pilocarpine administration to 0.6 ± 0.6 % during the dark period (p < 0.01), and decreased from 20.2 ± 19.9 % to 2.6 ± 2.6 % during the following light period (p < 0.01; Fig. 2a, b & c). Our previous results have shown that 10 Hz sham EA stimulation did not alter baseline EEGs [12]. We also observed that 10 Hz sham EA stimuli did not exhibit effect on pilocarpine-induced epileptiform EEGs (data not shown).

### CeA opioid receptors mediate EA’s effect on epilepsy

**Fig. 3:**
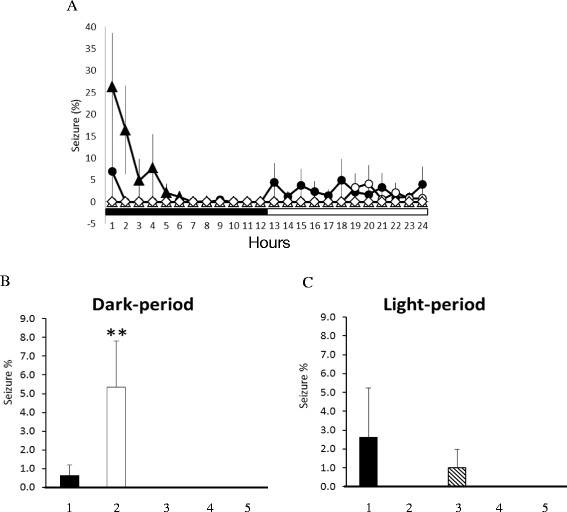
The summary of the effects of naloxone, naloxonazine, naltrindole and *nor*-binaltorphimine on the 10 Hz EA-induced suppression of epileptic activity. In panel **a**, the black circles represent the values obtained from the *PFS + EA + pilocarpine* group, the black triangles depict the results of the *naloxone + EA + pilocarpine* group, the white circles demonstrate the values obtained from the *naloxonazine + EA + pilocarpine* group, the white diamonds elucidate the results of the *naltrindole + EA + pilocarpine* group, and the white triangles indicate the data acquired from the *nor-binaltorphimine + EA + pilocarpine* group. Panel **b** depicts the results obtained from the dark period and panel C demonstrates the data acquired from the light period. Bars 1-5 spanning from the left to the right in panels **b** and **c** represent the results obtained from the *PFS + EA + pilocarpine* group, *naloxone + EA + pilocarpine* group, *naloxonazine + EA + pilocarpine* group, *naltrindole + EA + pilocarpine* group and *nor-binaltorphimine + EA + pilocarpine* group, respectively.**: p < 0.01 vs. the *PFS + EA + pilocarpine* group

Application of naloxone (10 μg) significantly blocked EA’s suppression effect on pilocarpine-induced epileptiform EEGs (Fig. 1d & Fig. 3). The average time epileptic EEGs were exhibited in the *naloxone + EA + pilocarpine* group was significantly enhanced to 5.4 ± 2.5 % during the dark period (p < 0.01 vs. *PFS + EA + pilocarpine* group; Fig. 3b). However, naloxonazine (10 μg), naltrindole (10 μg) and *nor*-binaltorphimine (10 μg) exhibited no effect on blocking EA’s effect (Fig. 3).

### Effects of administration of pilocarpine into the left CeA on sleep

**Fig. 4:**
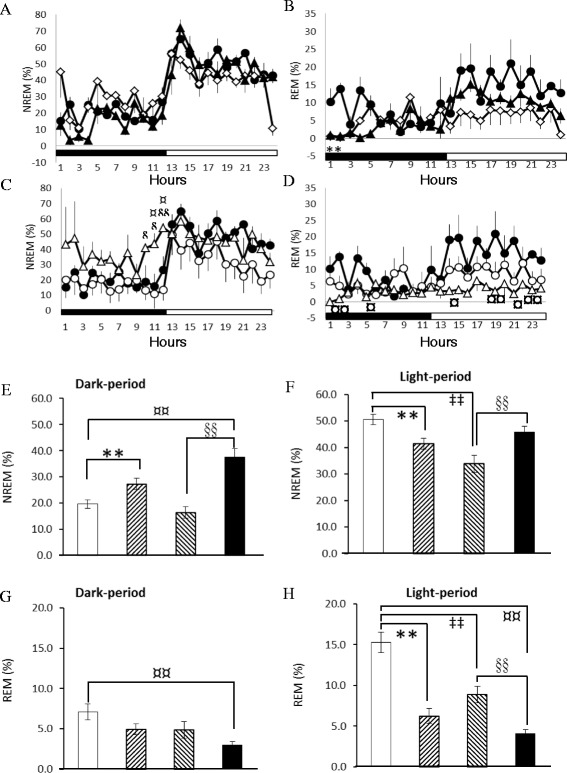
The effects of 10 Hz EA stimulation and pilocarpine in sleep. Panels **a & b**: NREM sleep and REM sleep obtained from the undisturbed baseline (naïve rats), the *sham EA* group and the *EA* group. Panels **c & d**: NREM sleep and REM sleep acquired from the baseline, the *pilocarpine* group and the *PFS + EA + pilocarpine* group. Black circles: the values obtained from undisturbed rats (baseline); black triangles: the values obtained from the *sham EA* group; open diamonds: the values acquired from the *EA* group; open circles: the values obtained from the *pilocarpine* group; open triangles: the data acquired from the *PFS + EA + pilocarpine* group. *: p < 0.05 vs. baseline. Black bar: the dark period; white bar: the light period. The bars from the left to the right in panels E-H represent the data acquired from the baseline, the *EA* group, the *pilocarpine* group and the *PFS + EA + pilocarpine* group. Panels **e** and **f** represent the percentage of time spent in NREM sleep during the 12-h dark period and the subsequent 12-h light period, respectively. Panels **g** and **h** indicate the percentage of time spent in REM sleep during the 12-h dark period and the subsequent 12-h light period, respectively. All symbols represent a significance with a p < 0.05 when comparing between two groups

Administration of pilocarpine into the CeA at the beginning of the dark period did not significantly change the amounts of NREM sleep and REM sleep during the dark period; however, both NREM sleep and REM sleep were significantly decreased during the following 12-h light period (Fig. 4c & d). The percentage of time spent in NREM sleep during the light period was decreased from 50.5 ± 1.9 % obtained from undisturbed rats to 33.8 ± 3.2 %, acquired after administration of pilocarpine (the *pilocarpine* group) at the dark onset (p < 0.05, Fig. 4f). The amount of REM sleep during the light period was also reduced from 15.3 ± 1.3 % to 8.9 ± 1.0 % (p < 0.05, Fig. 4h). These data are adapted from our previous observations in order to reduce the use of animals [22].

### Effects of 10 Hz EA stimuli on sleep in naïve and epileptic rats

There was no significant change in the sleep-wake activity when rats received sham EA stimuli (the *sham EA* group, Fig. 4a), except for sleep suppression of NREM and REM sleep during the first 4 h of the dark period. Our previous results have demonstrated that the anesthetization of rats for 30 min with ketamine, an NMDA receptor antagonist, prior to the dark period suppressed both NREM sleep and REM sleep during the first four hours of the dark period [12, 22]. Therefore, the decreases of NREM sleep and REM sleep during the first 4 h of the dark period when rats received the 10 Hz sham EA stimuli and were under anesthetization were primarily due to the effect of tiletamine, a composition of zoletil. In contrast, 10 Hz EA stimuli of Feng-Chi acupoints over three consecutive days enhanced physiological NREM sleep, but not REM sleep, during the dark period (the *EA* group) in the naïve rats. The percentage of time spent in NREM sleep during the dark period increased from 19.6 ± 1.7 % obtained from undisturbed rats to 27.3 ± 2.3 % (p < 0.05; Fig. 4). However, both NREM sleep and REM sleep were decreased in the following light period, which might simply be due to a compensatory effect. These results suggest that 10 Hz EA stimuli of bilateral Feng-Chi acupoints possess a somnogenic effect.

Rats received a 30-min 10 Hz EA stimulation of bilateral Feng-Chi acupoints over three consecutive days that blocked pilocarpine-induced NREM sleep reduction, but not the REM sleep reduction (Fig. 4c, d, f & h). The percentage of time spent in NREM sleep during the light period was enhanced to 45.8 ± 2.3 % (p < 0.05 vs. the *pilocarpine* group; Fig. 4f). Furthermore, 10 Hz EA stimuli also increased NREM sleep during the dark period in the epileptic rats; the time spent in NREM sleep increased from 16.5 ± 2.3 % obtained after pilocarpine administration to 37.5 ± 3.2 % (p < 0.05; Fig. 4c & e), although pilocarpine did not change any aspect of NREM sleep during the dark period.

### Effects opioid receptor antagonists on the 10 Hz EA-induced sleep alteration in epileptic rats

**Fig. 5:**
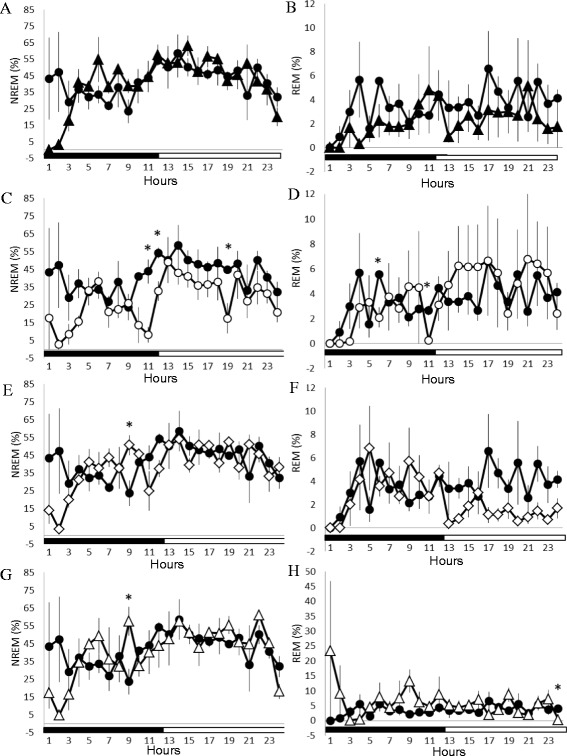
Naloxone, naloxonazine, naltrindole and *nor*-binaltorphimine on EA’s effect on sleep in rats with focal epilepsy. Panels **a & b**: the effects of naloxone on NREM sleep and REM sleep. Panels **c & d**: the effects of naloxonazine on NREM sleep and REM sleep. Panels **e & f**: the effects of naltrindole on NREM sleep and REM sleep. Panels **g & h**: the effects of *nor*-binaltorphimine on NREM sleep and REM sleep. Black circles: the data obtained from the *PFS + EA + pilocarpine* group; black triangles: the values acquired from the *naloxone + EA + pilocarpine* group; open circles: the results obtained from the *naloxonazine + EA + pilocarpine* group; open diamonds: the data acquired from the *naltrindole + EA + pilocarpine* group; open triangles: the values obtained from the *nor-binaltorphimine + EA + pilocarpine* group. *: p < 0.05 when comparing the values between two groups

**Fig. 6:**
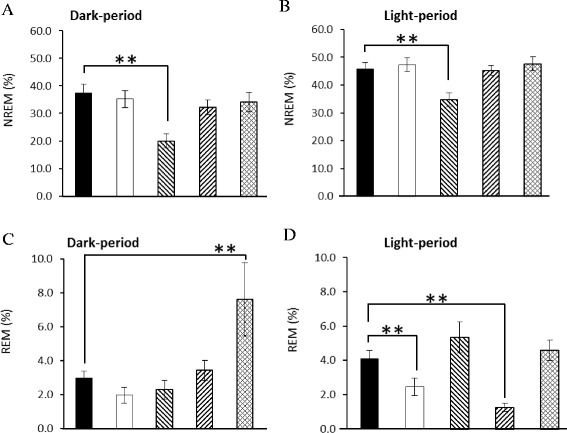
The summary of naloxone, naloxonazine, naltrindole and *nor*-binaltorphimine on EA’s effect on sleep in rats with focal epilepsy. The bars from the left to the right in each panel represent the data acquired from the *PFS + EA + pilocarpine* group, the *naloxone + EA + pilocarpine* group, the *naloxonazine + EA + pilocarpine* group, the *naltrindole + EA + pilocarpine* and the *nor-binaltorphimine + EA + pilocarpine* group. Panels **a** and **b** represent the percentage of time spent in NREM sleep during the 12-h dark period and the subsequent 12-h light period, respectively. Panels **c** and **d** indicate the percentage of time spent in REM sleep during the 12-h dark period and the subsequent 12-h light period, respectively. **: p < 0.05 when comparing the values between two groups

Administration of naloxone significantly blocked the 10 Hz EA-induced enhancement of NREM sleep during the first 3 h of the dark period in epileptic rats, but exhibited no effect during the light period (Fig. 5a & Fig. 6). The percentage of time spent in NREM sleep during the first 3 h of the dark period in the *naloxone + EA + pilocarpine* group was significantly decreased from 39.9 ± 11.0 % obtained from the *PFS + EA + pilocarpine* group to 7.08 ± 3.2 % (p < 0.05). REM sleep was not significantly altered after administration of naloxone (Fig. 5b & Fig. 6). Our previous study demonstrates that administration of naloxone into the CeA does not alter sleep activity in rats [22], suggesting that the EA-induced enhancement of NREM sleep is specifically mediated by CeA opioid receptors.

Administration of naloxonazine significantly blocked the 10 Hz EA-induced enhancement of NREM sleep during the dark period and subsequent light period in epileptic rats (Fig. 5c, Fig. 6a & b). The percentage of time spent in NREM sleep during the first 4 h of the dark period in the *naloxonazine + EA + pilocarpine* group was significantly decreased from 39.2 ± 8.3 % obtained from the *PFS + EA + pilocarpine* group to 11.1 ± 4.5 % (p < 0.05), and that of NREM sleep was reduced from 45.8 ± 2.3 % to 34.7 ± 2.5 % (p < 0.05) during the subsequent light period. REM sleep was not significantly altered after administration of naloxonazine (Fig. 5d & Fig. 6).

Administration of naltrindole significantly blocked the 10 Hz EA-induced enhancement of NREM sleep during the first 3 h of the dark period in epileptic rats, but demonstrated no effect during the light period (Fig. 5e & Fig. 6). The percentage of time spent in NREM sleep during the first 3 h of the dark period in the *naltindole + EA + pilocarpine* group was significantly decreased to 12.3 ± 3.5 % (p < 0.05 vs. the *PFS + EA + pilocarpine* group). REM sleep was not significantly altered after administration of naltrindole (Fig. 5f & Fig. 6).

Administration of *nor*-binaltorphimine significantly blocked the 10 Hz EA-induced enhancement of NREM sleep during the first 3 h of the dark period in epileptic rats, but demonstrated no effect during the light period (Fig. 5g & Fig. 6). The percentage of time spent in NREM sleep during the first 3 h of the dark period in the *nor-binaltorphimine + EA + pilocarpine* group was significantly decreased to 12.8 ± 4.3 % (p < 0.05 vs. the *PFS + EA + pilocarpine* group). No significant change occurred in REM sleep after administration of *nor*-binaltorphimine (Fig. 5H & Fig. 6).

## Discussion

Systemic or intracerebral administration of high-dose pilocarpine, a cholinergic muscarinic agonist, establishes the animal model of temporal lobe epilepsy and status epilepticus in rodents [39]. Several brain regions (e.g. the amygdala, thalamus, olfactory cortex, hippocampus, neocortex, and substantial nigra) are affected after pilocarpine administration [40]. As a matter of fact, the amygdala is the key brain structure that elicits epilepsy after administration of pilocarpine in rats or mice. Microinjection of bethanechol, a potent muscarinic agonist that is resistant to acetylcholinesterases, into the amygdala results in epileptiform EEGs, and the epileptic activities subsequently spread to the hippocampus and cortex [40]. Clinical and experimental evidence indicates that epilepsy and sleep reciprocally influence each other. It has been noticed that patients with different types of epilepsy experience different sleep disruptions. Patients with temporal lobe epilepsy (TLE) often encounter sleep fragmentation with a decrease in sleep efficiency, whereas patients with frontal lobe epilepsy (FLE) show little change in classical sleep parameters [41]. However, analysis of the microstructures of sleep in nocturnal FLE patients demonstrates sleep instability and arousal fluctuations [42]. In animal studies, we further elucidated that the occurrence of epilepsy at different zeitgeber time results in different sleep disturbances by altering either the homeostatic factors or the circadian rhythm of sleep-wake regulation [4, 5]. NREM sleep promotes seizure discharge [43], whereas REM sleep decreases seizure susceptibility [44]. Therefore, if a therapy can accomplish both epilepsy suppression and the improvement of sleep disturbance, it would be the most optimal for seizure control. EA stimulation of bilateral Feng-Chi acupoints may become a potential therapy to suppress epileptic activity and improve epilepsy-induced sleep disruptions. Although lacking scientific evidence, Lingshu Jing, an ancient Chinese literature, documents these indications of epilepsy suppression and insomnia treatment. The purpose of current study was therefore focused on the effect of 10 Hz EA stimuli of Feng-Chi acupoints on focal epilepsy, and the sleep disruptions in epileptic rats induced by administering a low dose of pilocarpine into the CeA. Our previous study had validated that the microinjection of low dose (1 mg/μl) pilocarpine into the left CeA induces focal epilepsy in rats. EEG signals were acquired from the left and right frontal, parietal and occipital cortices using multiple electrodes [21], and we confirmed this model again in current study. Our results indicated that no sleep parameter was altered during the dark period, but both NREM sleep and REM sleep were decreased in the following light period when pilocarpine was administered at the beginning of the dark period.

We examined the effect of 10 Hz EA stimuli of bilateral Feng-Chi acupoints in naïve rats and found that low frequency EA slightly induced epileptiform EEGs during the first hour after EA stimuli. This observation is consistent with our previous results with high frequency (100 Hz) EA stimuli of Feng-Chi [21]. The reason for this induction of epileptic activities after EA stimuli of Feng-Chi acupoints might be due to the nonspecific stimulation of the brainstem and raising EEG activities. Application of 10 Hz EA stimuli of Feng-Chi acupoints significantly suppressed pilocarpine-induced epileptiform EEGs, which differs from both the result of 10 Hz EA in naïve rats and the exacerbation effect of 100 Hz EA on epileptic activities [21]. Furthermore, 10 Hz of EA stimuli of Feng-Chi acupoints in naïve rats enhanced NREM sleep during the dark period, but decreased both NREM sleep and REM sleep in the subsequent light period. This observation differs from our previous finding that demonstrates 100 Hz EA stimuli of Feng-Chi acupoints exhibits no effect on physiological sleep in naïve rats [22]. This suggests that 10 Hz, but not 100 Hz, of EA stimuli of Feng-Chi acupoints promotes sleep. The decreases of NREM sleep and REM sleep in the subsequent light period might simply be due to the compensatory effect to counter the enhancement of NREM sleep during the dark period. We further found that the epilepsy-induced reduction of NREM sleep during the light period was blocked by 10 Hz EA of Feng-Chi acupoints. This result indicates that the effect of 10 Hz EA on the blockade of epilepsy-induced sleep reduction during the light period is mediated by a specific mechanism, since it exhibited an opposite action when 10 Hz EA was applied in the naïve rats. Based upon previous studies, we had hypothesized that EA of Feng-Chi acupoints stimulates vagus nerves and subsequently activates NTS [12, 13, 21, 22]. It is worthy to note from our current and past studies that different stimulation parameters of EA result in different outcomes in the regulations of EEG activity and sleep states. Consistent with the observations of EA stimuli, different parameters of the VNS can either induce electrographic synchronization or desynchronization. High-frequency dominant EEGs appear after cats received VNS with frequencies between 24-50 Hz [23]. Chase et al. have also demonstrated that desynchronized EEGs can be induced by lower frequency VNS (20 Hz), whereas high frequency (200 Hz) stimulation of vagus nerve causes EEG synchronization [45]. Fifty Hz VNS desynchronizes EEGs and blocks sleep spindles [24]. VNS increases both NREM sleep and REM sleep in the “encephale isole” cats [46], and enhances ponto-geniculate-occipital (PGO) wave density and the total amount of REM sleep in normal cats [47]. This might be because the vagus nerves contain A-, B- and C-fibers, which have different thresholds for VNS to excite the action potentials [48]. The acceptable therapeutic parameters for VNS are frequencies of 20-30 Hz with intensities of 0.5-3.5 mA; however, irreversible damage of the vagus nerve is caused by a stimulation frequency higher than 50 Hz [48]. Nevertheless, our current results depicted that 10 Hz EA of Feng-Chi acupoints can successfully suppress the epileptiform EEGs induced by pilocarpine and block pilocarpine-induced reduction of NREM sleep, which differs from the outcomes of 100 Hz EA on the exacerbation of both epilepsy and epilepsy-induced sleep disruptions [21, 22]. The choice of frequency for EA stimulation is an important issue in order to exert a therapeutic effect.

Endogenous opioid peptides (e.g., encephalin, β-endorphin, dynorphin and endormorphin) and their receptors (such as μ-, δ- and κ-opioid receptors) mediate most of the effects of acupuncture, especially in acupuncture-induced analgesia. Han and his colleagues have revealed that low-frequency (2 Hz) EA stimuli increase met-enkephalin, but not dynorphin, in the spinal cord, while high-frequency (100 HZ) EA stimuli increase the release of dynorphin rather than that of met-enkephalin [49]. We have previously demonstrated that μ-receptors in the NTS mediate the sleep enhancements induced by low-frequency (10 Hz) EA stimulation of bilateral Anmian acupoints, whereas the activation of κ-receptors contributes to the high-frequency (100 Hz) EA-induced sleep increases [12, 13]. Because the amygdala is the epileptic focus in the current animal model and plays an important role in the sleep regulation [50], we herein investigated the role of CeA opioid receptors. We found that the administration of naloxone, but not other specific opioid receptor antagonists, into the CeA blocked the effect of EA on epilepsy suppression, indicating the involvement of all types of opioid receptors. This finding is consistent with the results that indicate naloxone blocks the morphine-induced anticonvulsant effect [51]. Our results also demonstrated that the microinjection of naloxonazine into the CeA significantly blocks 10 Hz EA stimulation-induced NREM sleep disruption during the light period in rats with focal epilepsy, suggesting the role of μ-opioid receptors in the effects of 10 Hz EA stimulation. Furthermore, the NREM sleep enhancement induced by 10 Hz EA during the dark period in epileptic rats was also blocked by naloxone, naloxonazine, naltrindole and *nor*-binaltorphimine. Since pilocarpine did not alter NREM sleep during the dark period and the administration of naloxone into CeA exhibited no effect on sleep parameters in naïve rats [22], this blockade effect by opioid receptor antagonists is primarily due to the specific action of CeA opioid receptors on the EA-induced sleep.

## Conclusions

Our current results indicated that low-frequency (10 Hz) EA stimulation of bilateral Feng-Chi acupoints successfully suppresses pilocarpine-induced focal epilepsy and blocks sleep disruption in epileptic rats. Our current study also demonstrated the involvement of CeA opioid receptors in mediating the effects of EA stimulation.
